# Snapshot of Anti-SARS-CoV-2 IgG Antibodies in COVID-19 Recovered Patients in Guinea

**DOI:** 10.3390/jcm13102965

**Published:** 2024-05-17

**Authors:** Solène Grayo, Houlou Sagno, Oumar Diassy, Jean-Baptiste Zogbelemou, Sia Jeanne Kondabo, Marilyn Houndekon, Koussay Dellagi, Inès Vigan-Womas, Samia Rourou, Wafa Ben Hamouda, Chaouki Benabdessalem, Melika Ben Ahmed, Noël Tordo

**Affiliations:** 1 Institut Pasteur de Guinée, Conakry BP 4416, Guinea; mathieu.sagno@pasteur-guinee.org (H.S.); noel.tordo@pasteur.fr (N.T.); 2 Agence Nationale de Sécurité Sanitaire, Conakry BP 797, Guinea; oumardiassy82@gmail.com; 3 Centre Médico-Social de L’ambassade de France, Conakry BP 295, Guinea; jeanbaptistezogbelemou@gmail.com (J.-B.Z.); marilynhoundekon@gmail.com (M.H.); 4 Clinique Ambroise Paré, Conakry BP 1042, Guinea; 628961154jeanne@gmail.com; 5 Direction Internationale, Institut Pasteur, 75724 Paris, France; koussay.dellagi@pasteur.fr; 6 Institut Pasteur de Dakar, Dakar BP 220, Senegal; ines.vigan-womas@pasteur.sn; 7 Institut Pasteur de Tunis, Tunis BP 74-1002, Tunisia; samia.rourou@pasteur.tn (S.R.); : chaouki.benabdessalem@pasteur.tn (C.B.); melika.benahmed@gmail.com (M.B.A.)

**Keywords:** Guinea, COVID-19, SARS-CoV-2, ELISA, IgG antibodies, spike1 Receptor Binding Domain

## Abstract

**Background**: Because the regular vaccine campaign started in Guinea one year after the COVID-19 index case, the profile of naturally acquired immunity following primary SARS-CoV-2 infection needs to be deepened. **Methods:** Blood samples were collected once from 200 patients (90% of African extraction) who were recovered from COVID-19 for at least ~2.4 months (72 days), and their sera were tested for IgG antibodies to SARS-CoV-2 using an in-house ELISA assay against the Receptor Binding Domain (RBD) of the SARS-CoV-2 spike1 protein (RBD/S1-IH kit). **Results:** Results revealed that 73% of sera (146/200) were positive for IgG to SARS-CoV-2 with an Optical Density (OD) ranging from 0.13 to 1.19 and a median value of 0.56 (IC95: 0.51–0.61). The median OD value at 3 months (1.040) suddenly decreased thereafter and remained stable around OD 0.5 until 15 months post-infection. The OD median value was slightly higher in males compared to females (0.62 vs. 0.49), but the difference was not statistically significant (*p*-value: 0.073). In contrast, the OD median value was significantly higher among the 60–100 age group (0.87) compared to other groups, with a noteworthy odds ratio compared to the 0–20 age group (OR: 9.69, *p*-value: 0.044*). Results from the RBD/S1-IH ELISA kit demonstrated superior concordance with the whole spike1 protein ELISA commercial kit compared to a nucleoprotein ELISA commercial kit. Furthermore, anti-spike1 protein ELISAs (whole spike1 and RBD/S1) revealed higher seropositivity rates. **Conclusions:** These findings underscore the necessity for additional insights into naturally acquired immunity against COVID-19 and emphasize the relevance of specific ELISA kits for accurate seropositivity rates

## 1. Introduction

Coronavirus disease 2019 (COVID-19) entered Africa in mid-February 2020 through Egypt, while Central and West Africa were affected beginning from mid-March [1,2]. In the absence of robust health systems, most African countries restricted testing to priority groups, such as symptomatic, vulnerable, or hospitalized persons [3,4]. The COVID-19 burden in Africa was considered lower than elsewhere worldwide with mostly asymptomatic or mild infections and few reported deaths [5]. On 18 November 2022, the World Health Organization (WHO) reported 12.7 million confirmed cases and 257,984 deaths in Africa, corresponding to only 2% of the global burden [6]. In Guinea, after the index detection on 13 March 2020, 38,465 infected people and 468 deaths were reported up to 12 October 2023, corresponding to only a 1.2% lethality [7]. Apart from limited doses of a Russian vaccine (Sputnik V) that were available from late 2020, the vaccine campaign started at Conakry in March 2021 and in the rest of the country in April 2021 with the Sputnik V and Sinopharm (Chinese) vaccines [8]. New type of vaccines (Sinovac, Johnson & Johnson, Pfizer/BioNTech, Oxford-AstraZeneca, and Moderna vaccines) then arrived through the COVAX initiative from April 2021 [8]. In about the same period, it was estimated that around two-thirds of the African population had been exposed to SARS-CoV-2 and had developed a natural immune protection [9], despite seroprevalence studies that ranged from 19.7% to 26% in sub-Saharan Africa [10,11]. In Guinea, seroprevalence studies showed a 2.4-fold increase (17.3% to 42.4%) of anti-SARS-CoV-2 antibodies in the general population between December 2020 and June 2021 [12,13]. The pre-existing immunity from previous exposure to endemic viruses (including coronavirus) might have influenced the population immune status and favored COVID-19 disease control [14,15]. On the other hand, it was also reported that there were infected individuals with very weak antibody induction and/or those whose antibody levels rapidly waned over time [16]. The naturally acquired immunity against COVID-19 needs to be clarified.

From March 2020 to April 2021, the Institut Pasteur de Guinée (IPGui) tested more than 21,000 suspected patients for SARS-CoV-2 infection, resulting in 7600 positive samples (including control tests) stored at the IPGui Biobank (−80 °C). The present study describes the follow-up for about 200 patients infected during this period and more particularly the level and duration of their naturally acquired immunity against SARS-CoV-2. Three ELISA protocols were used and compared to evaluate the patients’ IgG response: the first was produced for the project and targeted the Receptor Binding Domain of the spike1 protein (RBD/S1-IH kit); the second and third were acquired commercially and were against the spike1 protein (S1-EU kit) and the nucleoprotein (NP-IV kit), respectively. The results are discussed with regard to demographic variables (age, sex), intensity (clinical symptoms, viral load), and the duration of positivity during the original infection as well as the duration of the post-infection period.

## 2. Materials and Methods

### 2.1. Study Design and Selection Criteria

This was a retrospective case study to evaluate the humoral response (IgG immunoglobulin) to naturally acquired SARS-CoV-2 infection in patients who subsequently recovered. Pregnant women, persons on immunosuppressive therapy, and those claiming to have been reinfected or vaccinated with any of the COVID-19 vaccines were excluded from this study. Among about 2500 patients infected between March 2020 and April 2021 who had their related data stored at the IPGui Biobank (date of testing, duration of positivity, cycle threshold values), 1000 persons meeting the selection criteria were subjected to the enrollment procedure and were contacted individually by phone in local dialects. After validation of the selection criteria and explication of the study objectives, 300 persons who denied ever being vaccinated expressed their interest and motivation to participate in this study. They received an invitation to visit the medico-social center of the French embassy with travel costs covered, and they were reminded about their appointment by SMS and direct call one week and one day in advance, respectively. On the day of inclusion, written informed consent was signed by each individual or, in the case of minors, their parents or legal guardians before enrollment. The participants were informed that all data collected would remain anonymous and be treated as confidential. Data privacy protection was also guaranteed by using anonymous samples (barcoding). Finally, 200 persons were accepted to participate in this study without incentives.

### 2.2. Data and Sample Collection

Demographic (age, sex) and molecular (CT values of RT-qPCR tests, duration of positivity) data available from the IPGui Biobank database were confirmed by a standardized face-to-face interview of each participant including socio-demographic characteristics, occupation, symptoms, disease outcome, and treatments. Whole blood samples were centrifuged at 2000× *g* for 20 min and serum was collected in barcoded tubes and stored at −80 °C in the IPGui Biobank.

### 2.3. Enzyme-Linked Immunosorbent Assay (ELISA)

The presence of SARS-CoV-2 specific IgG Ab in serum was assessed using three ELISA assays: one produced at the Institut Pasteur de Tunis [17] and two commercial kits.

#### 2.3.1. Anti-SARS-CoV-2-RBD/S1 IgG ELISA (RBD/S1-IH)

Nunc MaxiSorp™ 96-well microplates were coated with 100 ng/well of recombinant Receptor Binding Domain of the spike1 protein (RBD/S1 protein) (IP Tunis, Tunis, Tunisia) produced in SF9 cells [18] and incubated at 4 °C overnight. Plates were washed three times with PBS 1% Tween-20, then blocked with 5% skim milk in PBS-T for 1 h at 37 °C. After discarding the blocking buffer, the plates were washed three times with PBS, then 50 µL of each serum sample diluted at 1:400 in PBS-T was added in the wells and incubated for 2 h at 37 °C. After six washings with PBS-T, plates were incubated with 100 µL of 1:8000 diluted peroxidase-conjugated anti-human goat IgG for 1 h at 37 °C. After six washes, plates were revealed by adding 50 µL of horseradish peroxidase (HRP) chromogenic substrate [3,3′,5,5′-tetramethylbenzidine (TMB) (BD Biosciences, Franklin Lakes, NJ, USA, 555214)]. After 30 min, the reaction was stopped by adding 50 µL of sulfuric acid (2 N). The absorbance was monitored at 450/630 nm using a Multiskan™ FC Microplate Photometer. The cutoff value was estimated at 0.34 OD630, as the average OD of 50 pre-COVID human sera samples provided by the Institut Pasteur de Dakar plus 3 standard deviations (SDs). For each ELISA plate, positive and negative controls were used to validate the test. All serum samples were tested at least twice.

#### 2.3.2. Anti-SARS-CoV-2-S1 IgG ELISA Kit (S1-EU Kit) 

The Anti-SARS-CoV-2 ELISA assay (Cat # EI 2606-9601 G; Euroimmun, Lübeck, Germany) estimates the IgGs against the spike1 protein (IgG S1-EU kit). The assay and interpretation were performed on 1/101 diluted human sera according to the manufacturer’s instructions.

#### 2.3.3. Anti-SARS-CoV-2-NP IgG ELISA ID-Vet Kit (NP-IV Kit)

The ID Screen^®^ SARS-CoV-2-N IgG indirect ELISA assay (IDVet, Montpellier, France) estimates the IgGs against the nucleoprotein (NP-IV kit). The assays and interpretation were performed on 1/21 diluted human sera according to the manufacturer’s instructions.

### 2.4. Statistical Analysis

#### 2.4.1. Data Base Analysis and Variables

A descriptive statistical analysis was conducted using R studio software (Version 2023.06.0 + 421). Frequency and percentage estimated the qualitative variables while the quantitative variables were measured by mean, median with 95% confidence interval (IC95), and min–max ranges.

#### 2.4.2. Normality Tests of Quantitative Variables (Age, OD of ELISA, Time for Recovery)

The data distributions were first compared to normal or Gaussian distribution to determine the statistic test choices: parametric or non-parametric tests. This analysis was performed by visual inspection (histogram and Q-Q plot) and by significance tests (Kolmogorov–Smirnov (K-S) normality test and Shapiro–Wilk test (SW)).

#### 2.4.3. Test of Independence between Quality Variables: Pearson’s χ^2^ and Odds Ratio Values

The Pearson chi-square (χ^2^) test was used to estimate a possible association between each categorical/class variable (sex, age, post-infection period (PI period)) considered independently and the ELISA results. The strength of the association was estimated from a univariate logistic regression model using dichotomous ELISA status (1 = positive; or 0 = negative). The generalized linear models (glms) were built under the form “OD-ELISA (numeric) ~ term (linear predictor for response)” in dual combinations to obtain the odds ratio (OR) of positive versus negative in function of sex, age group, and post-infectious period using “female”, “0–20 years old”, and “0–3 months” as references, respectively. Statistical significance was considered when the *p*-value was < 0.05 with a confidence interval of 95% (IC95).

#### 2.4.4. Relation between Quantitative Variables

The Kruskal–Wallis test by ranks (KW) (same as one-way analysis of variance for non-normal distribution) was used to compare the distribution of continuous variables (i.e., age, OD median values of ELISA, duration of COVID-19 positivity, etc.). Statistical significance was considered when the *p*-value was < 0.05. 

### 2.5. ELISA Assays Comparison

#### 2.5.1. Correlation between ELISA Seroreactivity: Spearman Coefficient of Rank (Rho)

The strength and direction of association between two distributions was measured by the Spearman’s coefficient of rank correlation (Rho) with a confidence interval of 95% (IC95) as a number between −1 (perfect negative association), 0 (no association), and 1 (perfect positive association). The correlation coefficient was statistically significant when the *p*-value was < 0.05. The statistical analyses were performed using MedCalc Statistical Software version 20.215 for Windows (MedCalc Software, Ostend, Belgium).

#### 2.5.2. Concordance between ELISA Seropositivity: Percentage of Agreement and Kappa Statistic

The percentage of agreement scores between the ELISA assays were estimated using Cohen’s Kappa Statistic by taking account of chance agreement or the rater independence. Kappa statistics were estimated by binary classification (seropositive = 1 and seronegative = 0 according to cutoffs where uncertain results were not included). They were calculated from the observed and expected frequencies on the diagonal of a square contingency table. The K was interpreted as null (<0), light (0.00–0.20), fair (0.21–0.40), moderate (0.41–0.60), substantial (0.61–0.80), or almost perfect (0.81–1.00) agreement [19]. 

### 2.6. Ethical Considerations

This study complied with the Ethical Principles for Human Research Standards, as its protocol received clearance from the Guinean National Ethics Committee for Research in Health (N°109/CNERS/20).

## 3. Results

To evaluate the level and duration of the naturally acquired immunity against SARS-CoV-2 in the general population in Guinea, we performed a retrospective study among non-vaccinated patients who had recovered from COVID disease. From about 1000 individuals who had been diagnosed as “positive” by the Institut Pasteur de Guinée (IPGui) between March 2020 and April 2021, and whose data/samples had been collected in the IPGui Biobank, 300 individuals matching the selection criteria (no pregnant women, no immunosuppressive therapy, no vaccinees, no known new SARS-CoV-2 infection) were contacted individually by phone and expressed their interest in this study. Finally, 200 persons, at between 2.4 and 15 months after their recovery, accepted the invitation to visit the medico-social center of the French Embassy in Conakry to participate in this study. Each participant was submitted to a face-to-face questionnaire related to COVID-19 disease outcome (clinical symptoms, treatment, hospitalization) before blood sampling and further testing to evaluate the anti-SARS-CoV-2 antibody response.

### 3.1. Population Study Description

**Figure 1:**
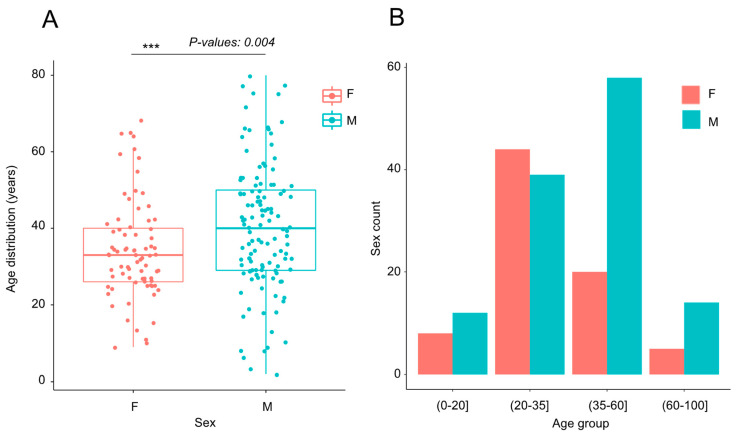
Age distribution by sex. (**A**) The horizontal lines of the boxplots show the median of ages values according to the sex: female (F) or male (M). The lower and the upper boundaries of the boxes indicate the 25th and the 75th percentiles, respectively. The Kruskal–Wallis (KW) test was used for one-way analysis of variance. *p*-value: 0.004 referred to a very strong significant statistic (*p*-value < 0.001 ***). (**B**) The barplots show female (pink) and male (blue) distributions according to the 4 age groups (0–20], (20–35], (35–60], and (60–100] years (x axis).

Sixty-one percent (61%) of the participants (122/200) were males, a ratio globally similar to that of the Guinean population who had been diagnosed as positive (64.2% males, October 2023; [7]) since the beginning of the pandemic. In terms of demographic data, 90% of the participants were African (180/200), including 172 Guineans, while 19 were European citizens and one was Colombian. Most of them had a regular professional activity (>80%), 33 were students, and 4 were retirees (Table 1). The ages were from 2 to 80 years old, with mean and median ages of 37.59 (IC: [35.56–39.86]) and 35 (IC: [32.85–37.15]) years old, respectively (Figure 1A and Table S1). However, females were significantly younger than males (KW = 8.14, *p*-value: 0.004 ***) with mean and median ages at 34.22 (IC: [34.78–40.65]) and 33 (IC: [30.06–35.93]) years old, respectively, compared to 39.9 (IC: [34.77–40.65]) and 40 (IC: [37.0–42.93]) years old for males (Figure 1A and Table S1). We defined four age groups (0–20, 20–35, 35–60, and 60–100), corresponding to 10% (20/200), 41.5% (83/200), 39% (78/200), and 9.5% (19/200) of the panel, respectively. The gender gap increased even more along this age distribution: a higher proportion of females and males were observed in the age classes below and above 35 years old, respectively, as visualized in the residual barplot (Figure 1B) and statistically validated (Pearson χ^2^ = 14.04; *p*-value: 0.00285 **).

### 3.2. COVID-19 Disease Description

Face-to-face individual interviews allowed us to collect patient declarations about their COVID-19 disease (Table 2). Thirty-five percent (35%: 70/200) declared ignorance of the contamination source, while the remaining 65% declared that they had been infected, in almost equal proportions, at home (21.5%: 43/200), at work (23%: 46/200), or during community events (20.5%: 41/200). Clinical symptoms were reported by 73% of the panel (145/200), mostly fatigue (80,7%: 117/145), followed by headache (75.9%: 110/145), sore throat and soreness (65.5%: 95/145), loss of taste and/or smell (58.6%: 85/145), diarrhea or insomnia (37.9%: 55/145), and nausea (27,5%: 40/145). The lock-down policy in Guinea led to the hospitalization of 74.5% of the participants (149/200). However, the percentage of treated patients was higher (92.5%: 185/200), suggesting that either they were sent back home with a treatment or that they performed self-medication (Table 2). 

### 3.3. SARS-CoV-2 Molecular Data

The viral load as assessed by a diagnostic RT-qPCR (cycle threshold—CT value) was recorded for 93.5% of the participants (187/200); the remaining 13 individuals were accompanying family members who declared the date of their infection but did not know their viral load. The original CT extended from 12 to 40, with mean 31.59 [30.52–32.66] and median 35 [33.95–36.07]). The duration of positivity (period between the first positive test and the first negative test) was recorded for 99.5% (199/200) of the participants and extended from 2 to 127 days (mean 16.69 days [14.82–18.56] and median 14 days [12.13–15.87]). The duration of 127 days was exceptional and observed only for one patient. The period between the COVID-19 diagnosis (first positive test) and the enrollment day (blood collection) or “post-infection” (PI) period was between 2.4 and 15.1 months (mean 9.89 months [9.31–10.47] and median 10.60 months [10.02–11.18]). For data analysis, five PI periods were distinguished: 0–3, 3–6, 6–9, 9–12, and 12–18 months, corresponding to 8% (16/200), 14.5% (29/200), 13% (26/200), 21% (42/200), and 42% (84/200) of participants, respectively.

### 3.4. Seroreactivity

**Figure 2:**
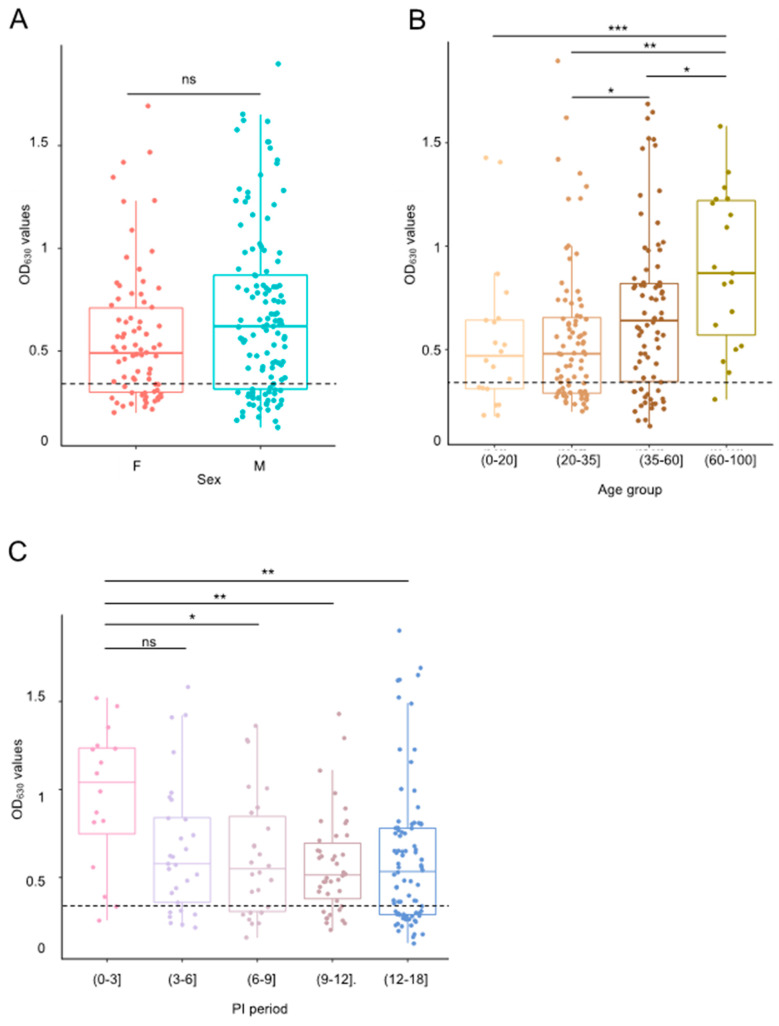
Seroreactivity by sex, age, and PI period. The horizontal lines of the boxplots show the OD median values according to (**A**) the sex: female (F) or male (M); (**B**) the 4 age groups: (0–20], (20–35], (35–60], and (60–100] years; or (**C**) the 4 PI periods: (0–3], (3–6], (6–9], (9–12], and (12–18] months. The lower and the upper boundaries of the boxes indicate the 25th and the 75th percentiles, respectively. The dashed lines represent the cutoff value of the ELISA assay (0.34). The Kruskal–Wallis (KW) test was used for one-way analysis of variance. *p*-value > 0.05 (ns), *p*-value < 0.05 *, *p*-value < 0.01 **, and *p*-value < 0.001 *** referred to, respectively, not significant, significant, very significant, and highly significant statistics.

The 200 sera from the COVID-19 recovered patients were tested twice using the RBD/S1-IH ELISA recognizing the Receptor Binding Domain of the spike1 protein. Specific IgG (OD values) was estimated according to the cut-off value (0.34). The global seropositivity frequency was 73% (146/200) for OD values ranging from 0.13 to 1.90 (mean 0.63 [0.58–0.69] and median 0.56 [0.51–0.61]). These values were almost the same for women and men, both in the seropositivity frequency, with 72% (56/78) and 74% (90/122) (OR:1.1, *p*-value: 0.8), and in OD median values with 0.49 and 0.62 (KW:3.2, *p*-values: 0.073), respectively (Table 3 and Table S2 and Figure 2A).

However, we observed significant differences in the distribution of OD median values between the age groups (KW: 15.73, *p*-values < 0.001 ***) (Table S2 and Figure 2B). Patients over 60 years of age clearly had the highest seropositivity frequency 95% (18/19, *p*-values: 0.044 *) and OD median value (0.87) among all age groups (*p*-values < 0.05 *) (Table 3 and Table S2). This tendency was still observable, although more slightly, in the 35–60 age group with seropositivity frequency at 76% and an OD median value of 0.68, significantly higher than the younger 20–35 (67% and 0.48) and 0–20 (65% and 0.47) age groups (Table 3 and Table S2). 

Concerning the duration of the SARS-CoV-2 antibodies in serum (post-infection period), it was clearly higher from 0 to 3 months post-infection with 94% (15/16) seropositivity frequency and a higher median value (OD 1.04, KW:11.49, *p*-values: 0.022 *) (Table 3 and Table S2 and Figure 2C). This was followed by a sudden decrease after 3 months which remained stable around OD 0.5 until 15 months post-infection (Table S2 and Figure 2C). Indeed, the participants tested within 3 months after their recovery showed at least a four-times better chance to be seropositive compared to the other participants, and about seven times better (OR: 0.14) compared to the 12–18 months period (Table 3).

### 3.5. Comparison of ELISA Assays

**Figure 3:**
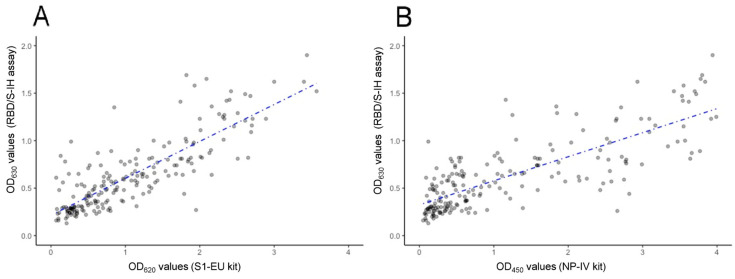
Correlation between SARS-CoV-2 ELISA assays. Scatter plots of Optical Density means at 630 nm (OD_630_) obtained by in-house ELISA kit against SARS-CoV-2 Receptor Binding Domain of the spike1 protein (RBD/S1-IH kit) (y-axis) and (**A**) Optical Density means at 620 nm (OD_620_) obtained by Euroimmun ELISA kit against SARS-CoV-2 spike1 protein (S1-EU kit) (x-axis), or (**B**) Optical Density means at 450 nm (OD_450_) obtained by ID-Vet ELISA kit against SARS-CoV-2 nucleoprotein (NP-IV kit) (x-axis), for 200 sera from the COVID-19 recovered patients. Blue dashed lines represent reduced major axis lines.

We compared the results obtained for the 200 sera (diluted 1:400) using the RBD/S1-IH assay targeting the Receptor Binding Domain of the SARS-CoV-2 spike1 protein with the results from two commercial ELISA kits: one against the complete spike1 protein (S1-EU kit; sera diluted 1:101) and the other against the nucleoprotein (NP-IV kit; sera diluted 1:21). The scatter plots comparing OD mean values are shown in Figure 3A,B, respectively. The Spearman rank–order analysis revealed a strong correlation coefficient (rho = 0.797, IC: 0.724–0.869) between the RBD/S1-IH assay and the S1-EU kit, with the points corresponding to individual tests being clustered around the oblique regression line (Figure 3A). The correlation was lower with the NP-IV kit, as expected when using a different nucleoprotein antigen, but still high and significant (rho = 0.760, IC: 0.690–0.831). According to the manufacturer’s cutoffs, the ELISA S1-EU kit detected 76.5% of positive sera (153/200), a result close to the 73% positive sera (146/200) detected with the RBD/S1-IH assay. However, only 60.5% (121/200) of the sera were positive with the NP-IV kit. In summary, the two ELISAs targeting complete or partial spike1 protein presented 87.5% concordance (kappa 0.669 (IC: 0.55–0.78; SE: 0.061) while only 81.5% concordance (kappa 0.571 (IC: 0.449–0.693, SE: 0.062) was observed between the ELISAs using the spike or the nucleoprotein as antigens.

## 4. Discussion

It is generally agreed that the IgG-seroconversion occurs from the second week after infection and persists over time to protect upon re-exposure to the same or a related virus. In this study, we investigated the naturally acquired humoral immune response of 200 COVID patients from the general population in Guinea 2.4 to 15 months after recovery from their SARS-CoV-2 primary infections which had occurred between April 2020 and April 2021. Negative controls consisted of pre-pandemic sera from a West African population negative for malaria [20]. ELISA results revealed that 76.5% (153/200) and 73% (146/200) of the patient sera recognized the full spike1 protein (commercial S1-EU kit) or its receptor binding domain (in-house RBD/S1-IH kit), respectively. This shows the similar performance of both ELISA assays, with the in-house version being economically affordable to measure antibody responses in the SARS-CoV-2-infected as well as vaccinated individuals from early at 2 weeks to a long time post-infection [21,22]. 

We did not observe a significant difference in IgG response between men and women but the global seroreactivity level of the population dropped about 3 months post-infection, then remained stable and above the threshold of seropositivity until 15 months post-infection. It is noticeable that it persisted longer and higher in the older age groups, such as the 60–100 age group, where it only declined 9 months post-infection, and to a lesser extent in the 35–60 age group. This result is coherent with one preliminary study showing a significantly higher seroprevalence among Guinean individuals >40 years and mostly >50 years old [12,13]. A similar observation was also made in Ghana [23]. As sub-Saharan Africa (SSA) has a long history of emerging and endemic infectious diseases, it is conceivable that prior exposure to another ß-coronavirus might promote cross-reactive immunity against SARS-CoV-2 and generate a stronger humoral response. Such cross-reactivity between SARS and MERS viruses was already observed pre-COVID in Sierra Leone, a neighboring country of Guinea [14]. Alternatively, repeated exposures to the same or different pathogens, and in particular the immunomodulation caused by malaria which is classically circulating in Guinea and its treatment, have been shown to sometimes lead to IgG false positivity [24,25]. It would be interesting to evaluate this combined immunogenicity by simultaneously assessing the seroreactivity against various antigens and pathogens using the Multiplex Microsphere Immuno-Assay (MMIA) based on the Luminex technology [26,27].

We did not investigate the SARS-CoV-2 neutralizing activity of the detected IgG antibodies, which is a critical factor for clinical efficacy and cure [28,29]. However, it must be noted that patient sera recognized very efficiently by ELISA the complete spike1 protein (76.5%) or its receptor binding domain (73%), with a “substantial” agreement (kappa 0.669), despite the fact that serum dilutions were 1/101 and 1/400 for the platform designs, respectively, which may induce different results, as outlined in previous publications [30,31]. The lower proportion of sera recognizing the N protein (60.5%) could be due to a better immunogenicity of the spike1 protein and a dominant proportion of the IgGs targeting the RBD, which probably inhibit SARS-CoV-2 entry into the cell. Even if less sera had detected the nucleoprotein, this lack in sensitivity can be compensated for by a better specificity, in particular during disease progression [32,33,34], since the nucleoprotein is globally less variable than the spike protein. In the present study, most of the patients (77%: 154/200) were infected with 20-A to 20-D SARS-CoV-2 mutants, prior to the appearance in January 2021 of the first Alpha variant of concern (VOC), and no participant was exposed to the following divergent Delta (May 2021) or Omicron (December 2021) VOCs [35].

No significant correlation was observed between the level of IgG antibodies and the viral load at the original diagnosis (CT value) nor with the duration of positivity. If the Ct-value thresholds are important biological data in COVID-19 management, their relevance as predictors for disease outcome remains debated [36,37,38]. It was previously demonstrated that patients may show a rapid decline in antibody production quickly after the onset of infection [39] and this is possibly the case for the patients we tested as negative (36%: 54/200). Others can take many months in resolving a SARS-CoV-2 infection and sometimes develop a wide range of persistent symptoms with potential comorbidities with other non-communicable diseases, resulting in a “Long COVID-19” or “Post-Acute Sequelae of COVID-19” (PASC) status [40,41]. Among the patients we followed, one went 18 weeks with a PCR positive status (CT < 40) and was still showing a substantial OD value (1.49) more than 10 months after recovery.

It is important to outline that this study was performed when anti-SARS-CoV-2 vaccines were not regularly available for the Guinean population and interviews confirmed that the studied cohort was not vaccinated before sampling. While the older and health workers are generally considered as priority populations for targeted vaccine campaigns [8], our study shows that the older had longer and more robust IgG responses than others. Although we cannot exclude that this older cohort selected elite immune responders, the data suggest that the Guinean active population should also be a priority target for vaccination, because they have more risk to be in contact with new variants due to their occupations and/or leisure activities [42]. If many questions are still pending in terms of the effectiveness of antibodies to confer immunity, vaccination remains the first line of defense against infectious diseases in endemic regions of West Africa, contributing to the improvement of primary and global health in populations.

## Figures and Tables

**Table 1:** Demographic data of the participants.

Variables	Class	Frequencies (n)	Percentages (%)
Sex	Female	78	39
	Male	122	61
Nationalities	Guinean	172	86
	African ^+^	8	4
	European	19	9.5
	Others	1	0.5
Occupation	Administrator	61	31
	Mechanic/Engineer	29	15
	Market workers	19	10
	Housekeeper/Cleaner	9	5
	Driver	10	5
	Police officer	7	4
	Health workers	20	10
	Professor	8	4
	Student	33	17
	Retiree	4	2
Total		200	100

^+^ Origin countries: Ivory Coast (3), Senegal (2), Benin (1), Tunisia (1), and Comoros (1).

**Table 2:** COVID-19 disease descriptions from face-to-face questionnaire.

Variables	Class	Frequencies (n)	Percentages (%)
Transmission source	Work	46	23
	Home	43	21.5
	Community	41	20.5
	Unknown	70	35
Symptoms	Yes	145	72.5
	No	55	27.5
Hospital	Yes	149	74.5
	No	51	25.5
Treatment	Yes	185	92.5
	No	15	7.5
Total		200	100

Kruskal–Wallis (KW) and confidence interval (IC_95_).

**Table 3:** Seropositivity by sex, age, and PI period.

		Total		Positive		Risk Factor		
Variables	Class	N	%	N	%	OR	IC_95_	*p*-Value
Sex	Female ^+^	78	39	56	72	—	—	—
	Male	122	61	90	74	1.1	0.58–2.08	0.8
Age group	(0–20] ^+^	20	10	13	65	—	—	—
	(20–35]	83	41.5	56	67	1.12	0.38–3.06	0.8
	(35–60]	78	39	59	76	1.67	0.56–4.73	0.3
	(60–100]	19	9.5	18	95	9.69	1.47–193	0.044 *
PI period	(0–3] ^+^	16	8.12	15	94	—	—	—
	(3–6]	29	14.50	22	76	0.21	0.01–1.35	0.2
	(6–9]	26	13.20	18	69	0.15	0.01–0.95	0.089
	(9–12]	42	21.32	33	79	0.24	0.01–1.48	0.2
	(12–18]	84	42.64	57	68	0.14	0.01–0.75	0.064

Number (N), odds ratios (OR), and confidence interval (IC_95_). **^+^** reference group. * *p*-value < 0.05.

## Data Availability

All data are incorporated into this article.

## Supplementary Materials

The following supporting information can be downloaded at: https://www.mdpi.com/article/10.3390/jcm13102965/s1, Table S1: Age by sex of the participants; Table S2: Seroreactivity by sex, age, and PI period.

## References

[1] WHO WHO COVID-19 Dashboard. https://covid19.who.int/.

[2] WHO-Africa Coronavirus (COVID-19): Situation Reports, WHO African Region. https://www.afro.who.int/health-topics/coronavirus-covid-19.

[3] WHO-Africa: Six in Seven COVID-19 Infections Go Undetected in Africa. https://www.afro.who.int/news/six-seven-covid-19-infections-go-undetected-africa.

[4] Dzinamarira T., Dzobo M., Chitungo I. (2020). COVID-19: A perspective on Africa’s capacity and response. J. Med. Virol..

[5] Vaughan A. We Don’t Know Why So Few COVID-19 Cases Have Been Reported in Africa. https://www.newscientist.com/article/2236760-we-dont-know-why-so-few-COVID-19-cases-have-been-reported-in-africa.

[6] WHO Weekly Epidemiological Update on COVID-19. 29 June 2021. https://www.who.int/publications/m/item/weekly-epidemiological-update-on-covid-19---29-june-2021.

[7] ANSS Rapport de Situation de la COVID-19 en Guinée. 9 October 2023, Sitrep N°1279. https://anss-guinee.org.

[8] ANSS Plan National de Déploiement de la Vaccination contre la COVID-19 de Guinée. Mars 2022. https://portail.sante.gov.gn/wp-content/uploads/2023/02/GUINEE_PNDV-COVID-19_Version-2022-_-VF2.docx.

[9] WHO-Africa Over Two-Thirds of Africans Exposed to Virus Which Causes COVID-19: WHO Study. www.afro.who.int/news/over-two-thirds-africans-exposed-virus-which-causes-covid-19-who-study.

[10] Usuf E., Roca A. (2021). Seroprevalence surveys in sub-Saharan Africa: What do they tell us?. Lancet Glob. Health.

[11] Nyawale H.A., Moremi N., Mohamed M., Njwalila J., Silago V., Krone M., Konje E.T., Mirambo M.M., Mshana S.E. (2022). High Seroprevalence of SARS-CoV-2 in Mwanza, Northwestern Tanzania: A Population-Based Survey. Int. J. Environ. Res. Public Health.

[12] Soumah A.A., Diallo M.S.K., Guichet E., Maman D., Thaurignac G., Keita A.K., Bouillin J., Diallo H., Pelloquin R., Ayouba A. (2022). High and Rapid Increase in Seroprevalence for SARS-CoV-2 in Conakry, Guinea: Results from 3 Successive Cross-Sectional Surveys (ANRS COV16-ARIACOV). Open Forum Infect. Dis..

[13] Diallo M.S.K., Amougou-Atsama M., Ayouba A., Kpamou C., Mimbe Taze E.D., Thaurignac G., Diallo H., Lamare N.B., Bouillin J., Soumah A.K. (2023). Large Diffusion of Severe Acute Respiratory Syndrome Coronavirus 2 after the Successive Epidemiological Waves, Including Omicron, in Guinea and Cameroon: Implications for Vaccine Strategies. Open Forum Infect. Dis..

[14] Borrega R., Nelson D.K.S., Koval A.P., Bond N.G., Heinrich M.L., Rowland M.M., Lathigra R., Bush D.J., Aimukanova I., Phinney W.N. (2021). Cross-Reactive Antibodies to SARS-CoV-2 and MERS-CoV in Pre-COVID-19 Blood Samples from Sierra Leoneans. Viruses.

[15] Lacroix A., Vidal N., Keita A.K., Thaurignac G., Esteban A., De Nys H., Diallo R., Toure A., Goumou S., Soumah A.K. (2020). Wide Diversity of Coronaviruses in Frugivorous and Insectivorous Bat Species: A Pilot Study in Guinea, West Africa. Viruses.

[16] Beaudoin-Bussieres G., Laumaea A., Anand S.P., Prevost J., Gasser R., Goyette G., Medjahed H., Perreault J., Tremblay T., Lewin A. (2020). Decline of Humoral Responses against SARS-CoV-2 Spike in Convalescent Individuals. mBio.

[17] Benabdessalem C., Hamouda W.B., Marzouki S., Faye R., Mbow A.A., Diouf B., Ndiaye O., Dia N., Faye O., Sall A.A. (2023). Development and comparative evaluation of SARS-CoV-2 S-RBD and N based ELISA tests in various African endemic settings. Diagn. Microbiol. Infect. Dis..

[18] Boumaiza M., Chaabene A., Akrouti I., Ben Zakour M., Askri H., Salhi S., Ben Hamouda W., Marzouki S., Benabdessalem C., Ben Ahmed M. (2023). Development of an Optimized Process for Functional Recombinant SARS-CoV-2 Spike S1 Receptor-Binding Domain Protein Produced in the Baculovirus Expression Vector System. Trop. Med. Infect. Dis..

[19] Landis J.R., Koch G.G. (1977). The measurement of observer agreement for categorical dat. Biometrics.

[20] Yadouleton A., Sander A.L., Moreira-Soto A., Tchibozo C., Hounkanrin G., Badou Y., Fischer C., Krause N., Akogbeto P., de Oliveira Filho E.F. (2021). Limited Specificity of Serologic Tests for SARS-CoV-2 Antibody Detection, Benin. Emerg. Infect. Dis..

[21] Villafane L., Vaulet L.G., Viere F.M., Klepp L.I., Forrellad M.A., Bigi M.M., Romano M.I., Magistrelli G., Fermepin M.R., Bigi F. (2022). Development and evaluation of a low cost IgG ELISA test based in RBD protein for COVID-19. J. Immunol. Methods.

[22] Yilmaz A., Turan N., Kocazeybek B.S., Dinc H.O., Tali H.E., Aydin O., Tali H.B., Yilmaz S.G., Konukoglu D., Borekci S. (2022). Development of in House ELISAs to Detect Antibodies to SARS-CoV-2 in Infected and Vaccinated Humans by Using Recombinant S, S1 and RBD Proteins. Diagnostics.

[23] Mensah B.A., Ndong I.C., Quashie P.K., Guichet E., Abuaku B., Effah-Baafi Y., Tapela K., Asiedu K., Appiedu-Addo S.N.A., Obbeng L.B. (2022). Population-based sero-epidemiological investigation of the dynamics of SARS-CoV-2 infections in the Greater Accra Region of Ghana. Sci. Rep..

[24] Idowu A.O., Omosun Y.O., Igietseme J.U., Azenabor A.A. (2023). The COVID-19 pandemic in sub-Saharan Africa: The significance of presumed immune sufficiency. Afr. J. Lab. Med..

[25] Vogl T., Leviatan S., Segal E. (2021). SARS-CoV-2 antibody testing for estimating COVID-19 prevalence in the population. Cell Rep. Med..

[26] Ayouba A., Thaurignac G., Morquin D., Tuaillon E., Raulino R., Nkuba A., Lacroix A., Vidal N., Foulongne V., Le Moing V. (2020). Multiplex detection and dynamics of IgG antibodies to SARS-CoV2 and the highly pathogenic human coronaviruses SARS-CoV and MERS-CoV. J. Clin. Virol..

[27] Galipeau Y., Greig M., Liu G., Driedger M., Langlois M.A. (2020). Humoral Responses and Serological Assays in SARS-CoV-2 Infections. Front. Immunol..

[28] Liu K.T., Han Y.J., Wu G.H., Huang K.A., Huang P.N. (2022). Overview of Neutralization Assays and International Standard for Detecting SARS-CoV-2 Neutralizing Antibody. Viruses.

[29] Wohlgemuth N., Whitt K., Cherry S., Kirkpatrick Roubidoux E., Lin C.Y., Allison K.J., Gowen A., Freiden P., Allen E.K., St Jude Investigative T. (2021). An Assessment of Serological Assays for SARS-CoV-2 as Surrogates for Authentic Virus Neutralization. Microbiol. Spectr..

[30] Nakashima K., Ishida M., Matsui H., Yoshida C., Nagai T., Shiraga M., Nakaoka H., Otsuka Y., Nakagama Y., Kaku N. (2022). Immunogenicity and safety of COVID-19 vaccine in lung cancer patients receiving anticancer treatment: A prospective multicenter cohort study. Hum. Vaccin. Immunother..

[31] Nakagama Y., Komase Y., Kaku N., Nitahara Y., Tshibangu-Kabamba E., Tominaga T., Tanaka H., Yokoya T., Hosokawa M., Kido Y. (2022). Detecting Waning Serological Response with Commercial Immunoassays: 18-Month Longitudinal Follow-up of Anti-SARS-CoV-2 Nucleocapsid Antibodies. Microbiol. Spectr..

[32] Tuaillon E., Bollore K., Pisoni A., Debiesse S., Renault C., Marie S., Groc S., Niels C., Pansu N., Dupuy A.M. (2020). Detection of SARS-CoV-2 antibodies using commercial assays and seroconversion patterns in hospitalized patients. J. Infect..

[33] Sun B., Feng Y., Mo X., Zheng P., Wang Q., Li P., Peng P., Liu X., Chen Z., Huang H. (2020). Kinetics of SARS-CoV-2 specific IgM and IgG responses in COVID-19 patients. Emerg. Microbes Infect..

[34] Beavis K.G., Matushek S.M., Abeleda A.P.F., Bethel C., Hunt C., Gillen S., Moran A., Tesic V. (2020). Evaluation of the EUROIMMUN Anti-SARS-CoV-2 ELISA Assay for detection of IgA and IgG antibodies. J. Clin. Virol..

[35] Grayo S., Troupin C., Diagne M.M., Sagno H., Ellis I., Doukoure B., Diallo A., Bart J.M., Kaba M.L., Henry B. (2022). SARS-CoV-2 Circulation, Guinea, March 2020–July 2021. Emerg. Infect. Dis..

[36] Sala E., Shah I.S., Manissero D., Juanola-Falgarona M., Quirke A.M., Rao S.N. (2023). Systematic Review on the Correlation between SARS-CoV-2 Real-Time PCR Cycle Threshold Values and Epidemiological Trends. Infect. Dis. Ther..

[37] Platten M., Hoffmann D., Grosser R., Wisplinghoff F., Wisplinghoff H., Wiesmuller G., Schildgen O., Schildgen V. (2021). SARS-CoV-2, CT-Values, and Infectivity-Conclusions to Be Drawn from Side Observations. Viruses.

[38] Patel R., Babady E., Theel E.S., Storch G.A., Pinsky B.A., St George K., Smith T.C., Bertuzzi S. (2020). Report from the American Society for Microbiology COVID-19 International Summit, 23 March 2020: Value of Diagnostic Testing for SARS-CoV-2/COVID-19. mBio.

[39] Pooley N., Abdool Karim S.S., Combadiere B., Ooi E.E., Harris R.C., El Guerche Seblain C., Kisomi M., Shaikh N. (2023). Durability of Vaccine-Induced and Natural Immunity Against COVID-19: A Narrative Review. Infect. Dis. Ther..

[40] Kim C., Chen B., Mohandas S., Rehman J., Sherif Z.A., Coombs K., Force R.M.P.T., Initiative R. (2023). The importance of patient-partnered research in addressing long COVID: Takeaways for biomedical research study design from the RECOVER Initiative’s Mechanistic Pathways taskforce. eLife.

[41] Proal A.D., VanElzakker M.B. (2021). Long COVID or Post-acute Sequelae of COVID-19 (PASC): An Overview of Biological Factors That May Contribute to Persistent Symptoms. Front. Microbiol..

[42] Msellati P., Sow K., Desclaux A., Cottrell G., Diallo M., Le Hesran J.Y., Harczi G., Alfa D.A., Toure A., Manigart O. (2022). Reconsidering the COVID-19 vaccine strategy in West and Central Africa. Lancet.

