# Supplementary material for: Snapshot of Anti-SARS-CoV-2 IgG Antibodies in COVID-19 Recovered Patients in Guinea
Source: J Clin Med. 2024 May 17;13(10):2965. doi: 10.3390/jcm13102965 (PMC11122401; doi:10.3390/jcm13102965)
Supplement: Supplementary file 1 [file jcm-13-02965-s001.zip › jcm-2951053-supplementary.pdf]

Supplementary Materials

Table S1. Age by sex of the participants.

	Mean [IC_{95}]	Min–Max	Median [IC_{95}]	KW	P-value
Female	34.22 [34.78-40.65]	9-68	33 [30.06-35.93]	8.14	0.004***
Male	39.9 [34.77-40.65]	2-80	40 [37.0-42.93]		
Total	37.59 [35.56-39.86]	2-80	35 [32.85-37.15]		

Table S2. Seroreactivity by sex, age, and PI period.

Variable	Class	Median	KW value	P-value
Sex	Female	0.49	3.2	0.073
	Male	0.62		
Age group	(0-20]	0.47	15.73	0.001***
	(20-35]	0.48		
	(35-60]	0.68		
	(60-100]	0.87		
PI period	(0-3]	1.040	11.49	0.022*
	(3-6]	0.580		
	(6-9]	0.550		
	(9-12]	0.515		
	(12-18]	0.535		

Kruskal–Wallis (KW).
